# Functional tug of war between kinases, phosphatases, and the Gcn5 acetyltransferase in chromatin and cell cycle checkpoint controls

**DOI:** 10.1093/g3journal/jkad021

**Published:** 2023-02-06

**Authors:** Qihao Liu, Lorraine Pillus, Emily L Petty

**Affiliations:** Department of Molecular Biology, University of California San Diego, 9500 Gilman Drive, La Jolla, CA 92093-034, USA; Department of Molecular Biology, University of California San Diego, 9500 Gilman Drive, La Jolla, CA 92093-034, USA; Department of Molecular Biology, University of California San Diego, 9500 Gilman Drive, La Jolla, CA 92093-034, USA

**Keywords:** checkpoints, yeast, kinase, acetyltransferase, phosphatase PP2A

## Abstract

Covalent modifications of chromatin regulate genomic structure and accessibility in diverse biological processes such as transcriptional regulation, cell cycle progression, and DNA damage repair. Many histone modifications have been characterized, yet understanding the interactions between these and their combinatorial effects remains an active area of investigation, including dissecting functional interactions between enzymes mediating these modifications. In budding yeast, the histone acetyltransferase Gcn5 interacts with Rts1, a regulatory subunit of protein phosphatase 2A (PP2A). Implicated in the interaction is the potential for the dynamic phosphorylation of conserved residues on histone H2B and the Cse4 centromere-specific histone H3 variant. To probe these dynamics, we sought to identify kinases which contribute to the phosphorylated state. In a directed screen beginning with *in silico* analysis of the 127 members of yeast kinome, we have now identified 16 kinases with genetic interactions with *GCN5* and specifically found distinct roles for the Hog1 stress-activated protein kinase. Deletion of *HOG1* (*hog1Δ*) rescues *gcn5Δ* sensitivity to the microtubule poison nocodazole and the lethality of the *gcn5Δ rts1Δ* double mutant. The Hog1–Gcn5 interaction requires the conserved H2B-T91 residue, which is phosphorylated in vertebrate species. Furthermore, deletion of *HOG1* decreases aneuploidy and apoptotic populations in *gcn5Δ* cells. Together, these results introduce Hog1 as a kinase that functionally opposes Gcn5 and Rts1 in the context of the spindle assembly checkpoint and suggest further kinases may also influence *GCN5*'s functions.

## Introduction

Eukaryotic genomes are packaged into the complex known as chromatin, which contains DNA and associated proteins. Dynamic modification, movement, and assembly of chromatin allow efficient organization and flexible genomic architecture to provide structural support and modulate accessibility. Such dynamic structural changes are indispensable for many essential cellular processes, including transcriptional regulation, cell division, and DNA replication and repair.

The basic repeat unit of chromatin is the nucleosome, containing two heterodimers of histones H2A, and H2B, a tetramer of histones H3 and H4, and a unit length of DNA wrapped around these core histones (Kornberg and Lorch, 1999; McGinty and Tan, 2015). Variant histones provide distinct functions, such the yeast H3 centromere-specific variant Cse4 (Choy *et al.* 2012). Histones undergo extensive post-translational modifications, mediated by chromatin modifying enzymes which are responsible for the covalent attachment and removal of small chemical or protein marks (Strahl and Allis 2000; Kouzarides 2007; Henikoff and Shilatifard 2011; Tessarz and Kouzarides 2014; Millan-Zambrano *et al.* 2022). These marks promote regulation of chromatin structure, interactions between histones and DNA, and recruitment of nonhistone proteins to specific locations in the genome (Nickel *et al.* 1989; Lu *et al.* 2008; Mishra *et al.* 2016), thereby contributing significantly to chromatin dynamics for cellular functions.

Enhancing their complexity, histone modifications do not function in isolation. Crosstalk between histone modifications adds a level of control to chromatin modifications. As one example, previous studies linked methylation of histone H3 residues to ubiquitination of H2B (Wyce *et al.* 2007). Dynamic ubiquitination and de-ubiquitination of H2B-K124 at promoters and within open reading frames modulate the accumulation of methylation on histone H3 throughout the transcribed region, ensuring optimal transcription. Such dynamic chromatin modifications and the enzymes that mediate them together form a complex network that regulates growth and mediates responses to stress conditions and nutrient availability. (Altheim and Schultz, 1999; Viéitez *et al.* 2020; Hsieh *et al.* 2022).

Among modifying enzymes, Gcn5 is a conserved lysine acetyltransferase (K/HAT) that modifies histones H2B and H3 (Grant *et al.* 1997; Zhang *et al.* 1998; Suka *et al.* 2001), while functioning as an enzymatic subunit of several distinct complexes (Grant *et al.* 1997; Eberharter *et al.* 1999; Sterner *et al.* 2002; Helmlinger *et al.* 2021). Since histone acetylation can disrupt interactions between the negatively charged DNA and the positively charged lysine residues, Gcn5-mediated histone acetylation is crucial for many processes, notably transcriptional regulation (Imoberdorf *et al.* 2006; Xue-Franzén *et al.* 2013), and responses to DNA damage (Pai *et al.* 2014). Gcn5 is also an important player in cell cycle progression. Gcn5-influenced histone gene expression and nucleosome assembly are crucial for DNA replication and chromatin organization during early stages of the cell cycle (Burgess and Zhang 2010; Gong *et al.* 2020). Indeed, previous research confirmed that *GCN5* mutants suffer slow progression through the G1/S phase transition (Petty *et al.* 2016). Later in the cell cycle, Gcn5 interacts with centromeres (Vernarecci *et al.* 2008) and regulates gene expression crucial for mitotic progression (Krebs *et al.* 2000). In addition, *GCN5* mutants display phenotypes such as sensitivity to microtubule destabilization and slow progression through the G2/M transition (Zhang *et al.* 1998; Howe *et al.* 2001; Vernarecci *et al.* 2008; Petty *et al.* 2018).

Whereas the functions and biological significance of Gcn5-mediated histone acetylation are well established, defining the significance of interactions between Gcn5 and other proteins remains an area of active investigation (Petty and Pillus, 2021). Genetic screens provide a powerful tool to identify factors that functionally interact with one another, revealing both positive and negative relationships. In *S. cerevisiae*, deletion of *GCN5* is tolerated but causes a variety of phenotypes (Cherry *et al.* 2012; Petty *et al.* 2016). By introducing altered gene dosage or additional mutations in a *gcn5Δ* mutant and selecting for alleviation of *gcn5Δ* phenotypes, we previously identified *RTS1*, a gene encoding a regulatory subunit of protein phosphatase 2A (PP2A) (Healy *et al.* 1991; Shu *et al.* 1997), as a high copy suppressor of a distinct set of *gcn5Δ* phenotypes (Petty *et al.* 2016).

Overexpression of *RTS1* in *gcn5Δ* mutants rescues temperature sensitivity, DNA damage sensitivity, poor growth on nonfermentable carbon sources and chromosome segregation defects (Petty *et al.* 2016). Notably, histone H2B threonine residue 91 (H2B-T91) is required for the Rts1–Gcn5 interaction. Whereas the nonphosphorylatable H2B-T91A mutant hinders the rescue of *gcn5Δ* phenotypes by *RTS1* overexpression, the phosphomimic H2B-T91D or H2B-T91E mutations cause lethality, strongly suggests that orchestrated dynamic histone phosphorylation is crucial for growth and stress-response functions in *gcn5Δ* mutant cells.

A closer look at the Gcn5–Rts1 interaction in the context of chromosome segregation indicated that deletion of *GCN5* abolishes the centromeric localization of Rts1, which occurs during the spindle assembly checkpoint to promote proper tension sensing and faithful chromosome segregation. A serine residue on the centromere-specific histone Cse4-S180 was found to play a role in Rts1 centromeric localization (Petty *et al.* 2018). The mutant *cse4-S180A* restores *Rts1* centromeric localization whereas the phosphomimetic mutant *cse4-S180EE* causes synthetic lethality in *gcn5Δ* (Petty *et al.* 2018). These results suggest that tightly regulated dynamic phosphorylation of Cse4 residues is crucial for proper response to nocodazole in *gcn5Δ* mutants.

**Fig. 1.**
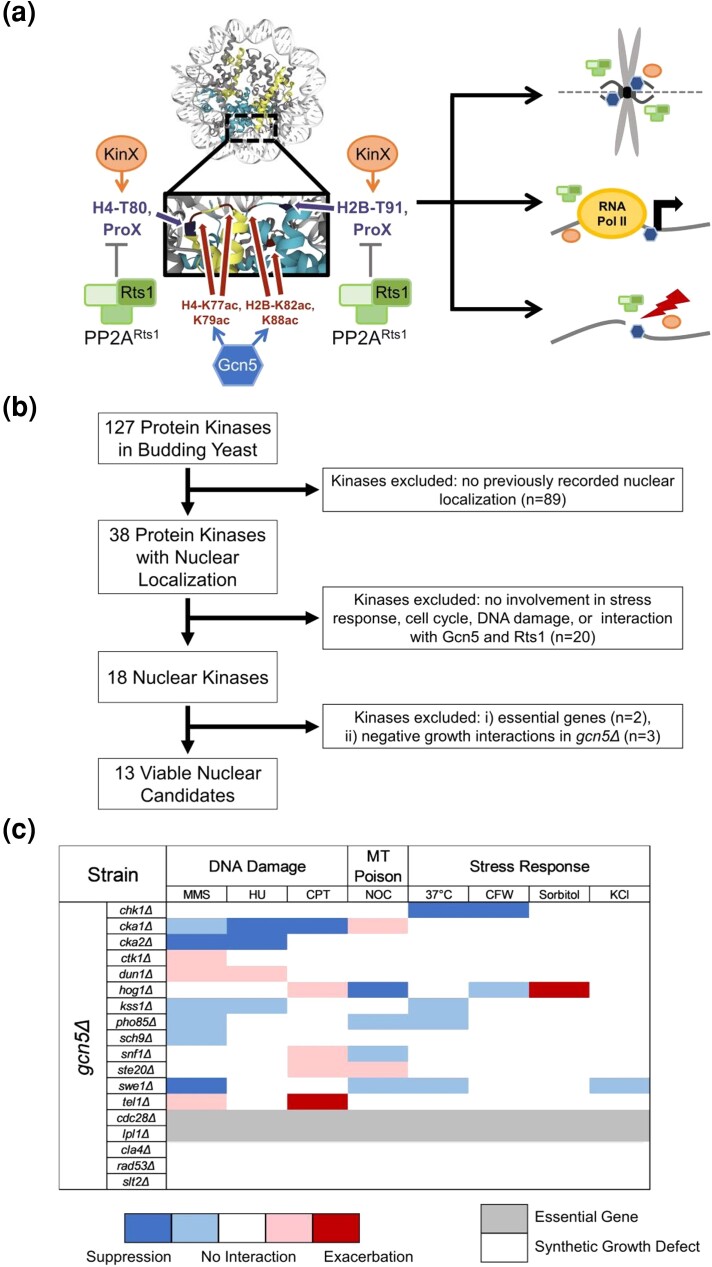
Identifying kinases that may function in the Rts1–Gcn5 interaction. a) Overexpression of the gene encoding a regulatory subunit of protein phosphatase 2A (PP2A, green), *RTS1*, rescues a variety of phenotypes caused by deletion of *GCN5* (dark blue) the gene encoding the catalytic histone acetyltransferase catalytic subunit of several multimeric complexes (Petty *et al.* 2016). Genetic screens demonstrated that specific H2B histone residues (blue) are important for the Rts1–Gcn5 interaction. Together, these data suggest that loss of Gcn5-mediated histone acetylation potentially disrupts the dynamic phosphorylation balance of specific histone residues which can be restored by the overexpression of *RTS1*. Thus, there may be a kinase or kinases (denoted as KinX) functioning at the *RTS1*–*GCN5* interaction nexus. The Kinase–Rts1–Gcn5 interaction nexus could take place in a variety of contexts, such as chromosome segregation, transcriptional regulation, and the DNA damage response. Further, these interactions may be mediated directly through histones or other protein substrates (ProX). We hypothesize that deletion of such a kinase would rescue the same set of *gcn5Δ* phenotypes suppressed by *RTS1* overexpression, whereas overexpression of the gene encoding the kinase may result in lethality in *gcn5Δ* cells. b) 13 kinases of interest were selected based on nuclear localization, cellular functions, interaction with *GCN5* and/or *RTS1*, and viability of the *gcn5Δ kinXΔ* double mutant. An *in silico* screen of the 127 member yeast kinome started with determination of nuclear localization as curated in the *Saccharomyces* Genome Database. Thirty-eight kinases were identified. Because *RTS1* overexpression specifically suppresses various *gcn5Δ* sensitivities the kinase candidates were further categorized based on their involvement in nutrient and stress-response, cell cycle progression, DNA damage, and interactions with *GCN5* and *RTS1*. Eighteen kinases met these criteria. Since the deletion of the nuclear kinase is hypothesized to rescue *gcn5Δ* phenotypes, the *gcn5Δ kinxΔ* double mutant must be viable. Therefore, two essential kinases were excluded. Of the remaining 16, published or preliminary results indicated that three caused severe sickness or lethality in *gcn5Δ* background, yielding 13 nuclear kinases for extended phenotypic and genetic analysis. c) Distinct patterns of phenotypic interaction with *gcn5Δ* are observed. A heat map to summarize the screen­­ results is shown. Semiquantitative dilution assays were performed with WT (LPY 5), *gcn5Δ* (LPY 13319), and the *gcn5Δ kinxΔ* mutant under a variety of stress conditions. Methyl methanesulfonate (MMS, 0.015 and 0.02%), hydroxyurea (HU, 0.05 and 0.1 M), and camptothecin (CPT, 3 μg/ml and 6 μg/ml) were used to induce distinct forms of DNA damage. Nocodazole (NOC, 1 μg/ml and 2 μg/ml) was used to induce chromosome segregation defects, calcofluor white (10 μg/ml) was used to produce cell wall-related stress, and 1 M KCl and sorbitol media were used to test for the sensitivity of salt and hyperosmolarity stress. The cells were scored after growth at 30°C for 3 days. A suppression of *gcn5Δ* phenotypes is shown as a blue cell in the heat map, with dark blue indicating strong suppression, and light blue indicating partial suppression. Exacerbation of *gcn5Δ* phenotypes is shown in red, with similar quantitative comparisons. When the *gcn5Δ kinxΔ* growth is comparable to *gcn5Δ,* a white cell is used to indicate no interaction. Dark and light gray cells denote essential gene deletions and gene deletions that cause lethality in the *gcn5Δ* background, respectively. The nonessential kinase deletions are shown in alphabetical order.

Kinases and phosphatases together create dynamic changes in histone phosphorylation states, exerting diverse effects on the structure and function of chromatin (Rossetto *et al.* 2012). Since the functional interaction between PP2A^Rts1^ and Gcn5 requires intact phosphorylatable residues, we hypothesized that a kinase may also function as part of a Rts1–Gcn5 interaction network. (Fig. 1a). Since overexpression of Rts1 potentially biases the chromatin phosphorylation balance toward the dephosphorylated state, loss of a kinase that shares a substrate with PP2A^Rts1^ might achieve the same effect. Therefore, the deletion of a kinase involved in the Rts1–Gcn5 interaction might suppress similar phenotypes in the *gcn5Δ* background as does overexpression of *RTS1.* To test this hypothesis, a screen considering all of the annotated yeast kinases in the yeast genome was performed.

By examining genetic interactions between kinase mutants and *gcn5Δ*, we have identified the Hog1 mitogen-activated protein kinase (MAPK) to be centrally involved in the Rts1–Gcn5 interaction. MAPKs are deeply conserved serine/threonine kinases in eukaryotes critical for signal transduction. In yeast, Hog1 functions in the high osmolarity glycerol (HOG) pathway and in governing cellular responses to changes in extracellular osmolarity (Brewster *et al.*,1993). When extracellular stresses are detected by membrane sensors, a cascade is activated, resulting in the phosphorylation and activation of Hog1 stress-response functions (Maeda *et al.* 1995; Posas *et al.* 1996; Maayan *et al.* 2012).

During the intracellular response to hyperosmotic stress, Hog1 interacts with cytoplasmic proteins (Bouwman *et al.* 2011), chromatin modifiers (Pérez-Martínez *et al.* 2020), and transcription factors (Rep *et al.* 1999, 2000), to modulate the concentration of intracellular osmolytes. Furthermore, Hog1 also interacts with components of cell cycle checkpoints to induce a transient cell cycle arrest at G1 (Escoté *et al.* 2004), S (Yaakov *et al.* 2009), G2 (Clotet *et al.* 2006), and M phases (Tognetti *et al.* 2020), until the stressful condition is resolved. Whereas the stress-response-related interactions of the MAPK and its partners are well documented in budding yeast, defining the functional role of MAPK chromatin modification and potential crosstalk remains an area of active investigation.

In this study, *hog1Δ* was identified as a suppressor of *gcn5Δ* mutants’ sensitivity to microtubule destabilization by nocodazole and the synthetic lethality of the *gcn5Δ rts1Δ* double mutant. The previously pinpointed histone H2B-T91 residue was required for the Hog1–Gcn5 interaction, further indicating that Hog1 and PP2A^Rts1^ together mediate dynamic modifications crucial for *gcn5Δ* viability. Cell cycle analysis suggests that *hog1Δ* alleviates defective cell cycle progression in cells lacking *GCN5* under microtubule destabilizing conditions. Together, these results point to a functional interaction between Hog1, Gcn5, and PP2A^Rts1^, in which Hog1 activity opposes Gcn5 and PP2A^Rts1^ in the context of the spindle assembly checkpoint.

## Materials and methods

### Yeast methods

Standard protocols for yeast growth were used (Guthrie and Fink, 1991). All yeast strains were grown at 30°C, except where indicated. For dilution assays, yeast cultures were adjusted to 1.0 OD_600_/ml and 1:5 serial dilutions were plated. Plates were photographed after 2–5 days incubation, as indicated in the figure legends. To test the effects of specific histone mutations, histone shuffle strains were constructed, with the WT histone shuffle strain (LPY 14461 *hht1-hhf1*Δ::*kanMX hta1-htb1*Δ::*natMX hta2-htb2*Δ::*HPH*) crossed to *hog1*Δ *gcn5*Δ (LPY 21367) to produce the *hog1Δ hht1-hhf1*Δ::*kanMX hta1-htb1*Δ::*natMX hta2-htb2*Δ::*HPH (LPY* 23129) and the *hog1Δ gcn5Δ hht1-hhf1*Δ::*kanMX hta1-htb1*Δ::*natMX hta2-htb2*Δ::*HPH* (LPY 23128) strains. Mutant *htb1* plasmids (*htb1-T94A, htb1-T91E*, or *htb1-T91D*) were transformed into the histone shuffle strains containing a *URA3*-WT histone plasmid. Strains used are listed in Supplementary Table 1. Yeast transformations were performed with either lithium acetate-based or simple single colony protocols. Plasmids used are listed in Supplementary Table 2.

Cells analyzed for budding index were taken from log-phase cultures and fixed with 70% cold ethanol. In independent experiments, more than 500 cells were examined for each variable.

### Media preparation

Yeast media were prepared with standard protocols, with modifications as noted (Guthrie and Fink, 1991; Sherman, 2002). Hydroxyurea (HU) plates were prepared by adding filter sterilized 1 M HU stock solution to synthetic complete (SC) media to a final concentration of 0.1 M or 0.05 M. Methyl methanesulfonate (MMS) plates were prepared at final concentrations of 0.01–0.025% in SC. Camptothecin (CPT) was added from a 3 mg/ml stock dissolved in DMSO to Yeast extract Peptone Adenine Dextrose (YPAD) media potassium phosphate buffered to pH 7.5 to a final concentration of 3–12 µg/ml. DMSO plates were prepared as a solvent control. Nocodazole (NOC) plates were prepared by adding 10 mg/ml of nocodazole dissolved in DMSO to YPAD to a final concentration of 1–2 µg/ml. KCl and sorbitol plates were prepared by adding filter sterilized KCl or sorbitol solutions to YPAD media to a final concentration of 1 M KCl or 1 M sorbitol. 5-FOA plates were prepared by adding filter sterilized 5-FOA solution to SC media to a final concentration of 0.5 × (∼5.7 mM). 0.5 × of standard 5-FOA concentrations was used due to increased sensitivity in the *gcn5Δ* background. We note that variable concentrations of nocodazole and 5-FOA were used in some experiments, due to the significantly differing sensitivities of wildtype and mutant strains. Concentrations used are noted for individual experiments in the figure legends.

### Flow cytometry

Samples were taken from exponentially growing cell cultures and fixed with cold 70% ethanol at 4°C overnight. Following fixation, the samples were treated with RNAse A (1 mg/ml) at 37°C overnight and stained with propidium iodide (PI) at 4°C overnight. All experiments used a BD ACCURI C6 flow cytometer for analysis. Fluorescence was detected with the FL2A channel with fluidics set to slow and an event limit of 30,000 events. During measurement, collected data were visualized in the BD ACCURI C6 flow cytometer histogram software. Microsoft Excel was used for statistical analysis and presentation. For analysis of sub-G1 populations, significance was evaluated with single-factor ANOVA. The significance of the data in 4(c) is *P* < 0.05 for all comparisons.

### Nocodazole sensitivity assay

10 mg/ml nocodazole dissolved in DMSO was added to log-phase cultures for a final concentration of 1–2 µg/ml. Samples were collected every hour for 6–8 hours following the nocodazole treatment. The samples collected were analyzed with flow cytometry as described above.

### CRISPR mutagenesis

The *hog1-T174A* catalytic mutation was introduced via CRISPR-mediated mutagenesis in a protocol adapted from Ran *et al.* (2013). CRISPR Direct (https://crispr.dbcls.jp/) was used for the identification of PAM sequences and guide RNA location within the *HOG1* sequence. The plasmid pML104, which encodes the Cas9 protein (a gift from L. McDonnell, UCSD), was digested with BclI and SwaI. Oligonucleotides containing a BclI 5′ overhang, *HOG1* gRNA sequence, and Cas9 scaffold sequence were hybridized and subsequently ligated into pML104. The homology directed repair template was synthesized via PCR with a pair of overlapping oligonucleotides containing the *hog1-T174A* mutation and the PAM disrupting silent mutation. The PAM (NGG) sequence was disrupted with a silent mutation, and the newly introduced codon at the PAM site and on threonine 174 were confirmed to be one of the preferred codons for yeast. The constructed CRISPR plasmid (1 µg) and the HDR templates (200 ng) were transformed into yeast cells via the lithium acetate method. *hog1-T174A* cells were selected based on positive growth on URA– media and a lack of growth on 1 M sorbitol media. The genotype was confirmed by sequencing PCR amplified products from genomic DNA (adapted from Hoffman and Winston, 1987). Oligonucleotides used for CRISPR mutagenesis are presented in Supplementary Table 3.

## Results

### A genetic screen identifies kinases with potential interactions with *GCN5*

To identify kinases that might function in the *GCN5*–*RTS1* interaction, we first surveyed the 127 genes encoding protein kinases (Rubenstein and Schmidt, 2007) in budding yeast via the *Saccharomyces* Genome Database (Fig. 1b). Considering that the *GCN5–RTS1* nexus involves specific histone residues (Petty *et al.* 2016), we hypothesized that a kinase participating in this interaction was likely to include nuclear functions. Of the yeast kinases, 38 genes encoding nuclear kinases were identified. Analysis of curated information for kinases with functions in DNA damage repair, cell cycle progression, stress/nutrient responses, and interaction with Gcn5 and Rts1 identified 18 candidate genes meeting these criteria (denoted as *KINX*) (Fig. 1b): *CDC28, CHK1, CKA1, CKA2, CLA4, CTK1, DUN1, HOG1, IPL1, KSS1, PHO85, RAD53, SCH9, SLT2, SNF1, STE20, SWE1,* and *TEL1* (Cherry *et al.* 2012).

To test for functional interactions with *GCN5*, classical genetic analysis was performed in which a double mutant was constructed with *gcn5Δ* and the nonessential genes encoding the nuclear kinase candidates (denoted as *gcn5Δ kinxΔ*). Diploids were constructed with the *gcn5Δ* parent containing a *CEN-URA3-GCN5* plasmid. After sporulation and dissection, all viable spores were genotyped. Double mutants were identified and to confirm the viability of *gcn5Δ kinxΔ* strains prior to phenotypic analysis, they were plated on medium containing 5-Fluoroorotic acid (5-FOA), which is used for robust counter-selection against *URA3* expression (Boeke *et al.* 1987). Growth on 5-FOA thus indicates that the double mutant is viable as it can survive without the *CEN-URA3-GCN5* plasmid. Since *CDC28* and *IPL1* are themselves essential in yeast, the corresponding double null mutants were not included in the initial screen (Fig. 1c). Furthermore, based on preliminary results and available literature pointing to synthetic lethalities and sickness between loss of Gcn5 and the deletion of *CLA4*, *RAD53*, or *SLT2*, analysis of those double mutants was not pursued (Tong *et al.* 2004; Burgess *et al.* 2010). Results from the test for synthetic lethality yielded 13 gene deletions were tolerated in the *gcn5Δ* background: *chk1Δ, cka1Δ, cka2Δ, ctk1Δ, dun1Δ, hog1Δ, kss1Δ, pho85Δ, sch9Δ, snf1Δ, ste20Δ, swe1Δ, and tel1Δ* (Fig. 1b and c). All of the kinases were categorized in three broad functional classes: (1) genes encoding cell cycle kinases, (2) genes encoding nutrient and stress-response kinases, and (3) genes encoding transcription and DNA damage-related kinases (Table 1).

**Table 1.** Genes encoding 18^a^ nuclear kinases of interest are classified into three groups based on major cellular functions.

**Cell Cycle**	*PHO85, SWE1, CHK1,* (*CDC28, IPL1, CLA4*)
**Nutrient/Stress Response**	*SNF1, SCH9, KSS1, HOG1, STE20, (SLT2)*
**Transcription/DNA Damage**	*CTK1, DUN1, CKA1, CKA2, TEL1, (RAD53)*

Note that five kinases, in parentheses, were not characterized further due to their essential nature or published or preliminary data indicating negative synthetic interactions with *gcn5Δ*.

Any rescue of *gcn5Δ* phenotypes by deletion of a gene encoding a nuclear kinase implies a potential interaction between that kinase and Gcn5. The phenotypes of the double mutants were therefore evaluated with various challenges, including elevated growth temperature, hyperosmolarity, DNA damage induction, and compounds that interfere with cell cycle progression (Petty *et al.* 2016). Figure 1c summarizes the results of this phenotypic survey. Deletions of *CKA1*, *CKA2*, *SWE1*, and *KSS1* were identified as suppressors of *gcn5Δ* mutant's sensitivity to DNA damage induced by hydroxyurea (HU), methyl methane sulfonate (MMS), and camptothecin (CPT) (Fig. 1c). *chk1Δ* was found to improve *gcn5Δ*'s growth at elevated temperatures and increased resistance to calcofluor white (CFW), a compound that causes cell wall damage (Fig. 1c). Among the mutants, *hog1Δ* had the strongest rescue of *gcn5Δ* sensitivity to nocodazole, a compound which can cause chromosome segregation defects (Fig. 1c). Like *RTS1* overexpression, which restores a variety of *gcn5Δ* phenotypes, deletion of the nuclear kinases resulted in unique patterns of resistance and sensitivity, indicating that multiple kinases may contribute to distinct aspects of Gcn5 function.

### Hog1 and the Rts1–Gcn5 interaction is linked to spindle function

**Fig. 2.**
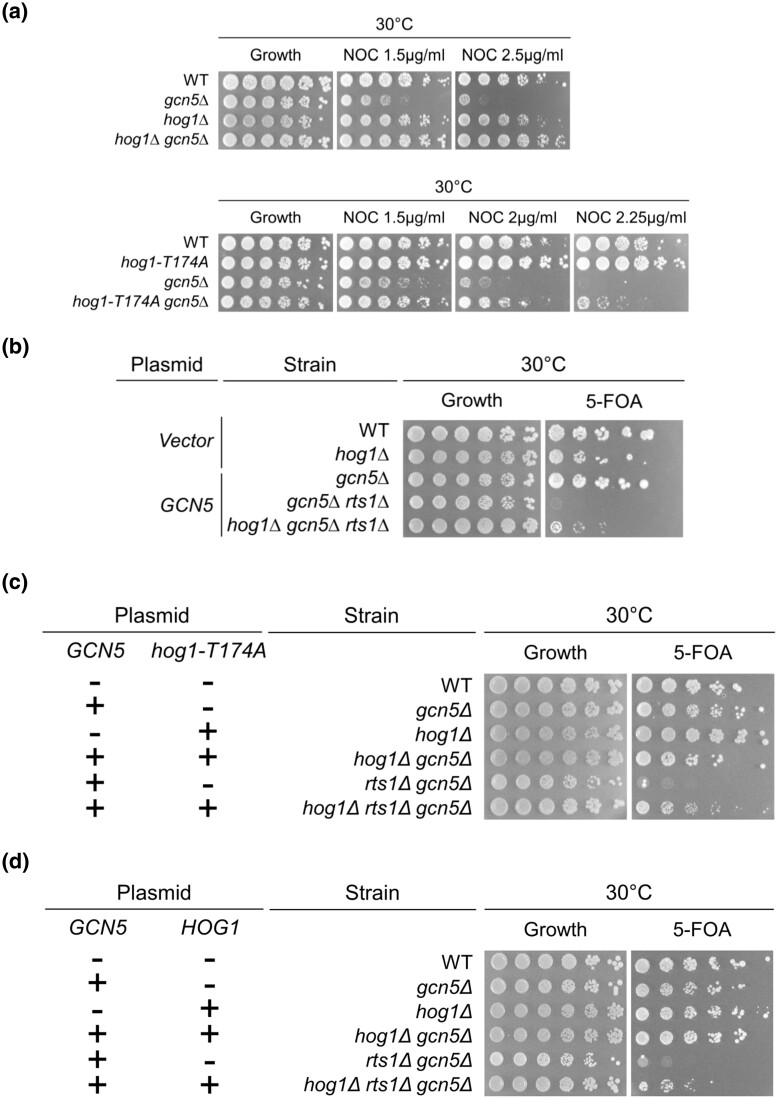
Deletion of *HOG1* or loss of its catalytic activity rescues *gcn5Δ* sensitivity to the microtubule poison nocodazole. a) The *hog1Δ gcn5Δ* strain shows increased resistance to nocodazole, relative to *gcn5Δ.* Dilutions of wildtype (LPY 5), *gcn5Δ* (LPY 13319)*, hog1Δ* (LPY 21375), *hog1-T174A* (LPY 23150), *hog1Δ gcn5Δ* (LPY 21367), and *hog1-T174A gcn5Δ* (LPY 23147) were plated onto YPAD (Growth) and nocodazole (NOC), the concentration of which ranges from 1.5 µg/ml to 2.25 µg/ml. Cells were grown at 30°C for three days. *b) hog1Δ* suppresses *rts1Δ gcn5Δ* synthetic lethality. The *hog1Δ gcn5Δ rts1Δ* triple mutant was constructed with a *CEN-URA3-GCN5* (pLP 1640) covering plasmid, WT and *hog1Δ* were transformed with a *CEN-URA3* vector control (pLP 126). Dilutions of wildtype (LPY 5 + pLP 126), *hog1Δ* (LPY 21375 + pLP126), *gcn5Δ* (LPY 13319 + pLP 1640)*, gcn5Δ rts1Δ* (LPY 15178 + pLP 1640), and *hog1Δ gcn5Δ rts1Δ* (LPY 23189 + pLP 1640) were plated onto URA- (growth) or 5-FOA to select against the *CEN-URA3-GCN5* plasmid and incubated at 30°C for three days. c) Loss of Hog1 catalytic activity suppresses *gcn5Δ rts1Δ* synthetic lethality. Wildtype (LPY 5), *hog1Δ* (LPY 21375), *gcn5Δ* (LPY 13319)*, hog1Δ gcn5Δ* (LPY 21367)*, gcn5Δ rts1Δ* (LPY 15178), and *hog1Δ gcn5Δ rts1Δ* (LPY 23189) containing either the *CEN-URA-GCN5* (pLP 1640) covering plasmid, or the *CEN-URA3-vector* (pLP 126) were transformed with either the *CEN-HIS3-hog1-T174A* plasmid or the *CEN-HIS3-vector*. Strains were plated onto URA-HIS- (growth) and HIS- 5-FOA to select against the *CEN-URA3-GCN5* plasmid. Cells were incubated at 30°C for three days. The *hog1Δ gcn5Δ rts1Δ* triple mutant containing *CEN-HIS3-hog1-T174A* showed growth on 5-FOA*. d) WT-HOG1* gene restores synthetic sickness in the *hog1Δ rts1Δ gcn5Δ* strain*. The CEN-HIS3-HOG1* plasmid or the *CEN-HIS3-vector* control were transformed into WT (LPY 5), *hog1Δ* (LPY 21375), *gcn5Δ* (LPY 13319)*, hog1Δ gcn5Δ* (LPY 21367)*, gcn5Δ rts1Δ* (LPY 15178), and *hog1Δ gcn5Δ rts1Δ* (LPY 23189) containing either the *CEN-URA-GCN5* (pLP 1640) covering plasmid, or the *CEN-URA3-vector* (pLP 126). Transformants were plated onto URA-HIS- (growth) and HIS-5-FOA and incubated at 30°C for three days. The *hog1Δ gcn5Δ rts1Δ* triple mutant containing the *CEN-HIS3-HOG1* showed reduced growth compared to the triple mutant containing the *CEN-HIS3-vector*.

Previous results demonstrated that increased *RTS1* dosage restores growth in nocodazole to strains lacking *GCN5*, linking the Rts1–Gcn5 interaction to spindle assembly checkpoint functions (Petty *et al.* 2018). In this study, we tested the growth phenotypes of the *gcn5Δ kinxΔ* double mutants on medium containing nocodazole. As noted above, deletion of *HOG1* was observed to significantly improve growth of *gcn5Δ* mutants under microtubule destabilizing conditions (Fig. 2a, top). We next tested if loss of Hog1 catalytic activity is the factor that underlies the rescue. The catalytically inactive Hog1 mutant was constructed by replacing the threonine 174 residue on the Hog1 activation loop with alanine via CRISPR-based mutagenesis. The *hog1-T174A* mutation prevents the activation of Hog1 catalytic activity by the upstream MAPKK (Maeda *et al.* 1995; Maayan *et al.* 2012). The *hog1-T174A gcn5Δ* double mutant was constructed genetically and tested for growth on nocodazole. Indeed, loss of Hog1 catalytic activity is sufficient to restore growth in the *gcn5Δ* background upon the nocodazole challenge (Fig. 2a, bottom). Further, loss of Hog1 catalytic activity improves nocodazole resistance not only in *gcn5Δ* mutants, but also has a modest effect in otherwise wildtype (WT) cells (Fig. 2a, bottom). Suppression of *gcn5Δ* sensitivity to nocodazole was somewhat weaker in *hog1-T174A gcn5Δ* when compared to the *hog1Δ gcn5Δ* mutant, an effect likely due to disruption, but not complete loss of catalytic activity in the point mutant. Overall, these results suggest that compromising Hog1 kinase activity generally reduces nocodazole sensitivity.

Concurrent loss of Gcn5 and Rts1 induces synthetic lethality (Petty *et al.* 2016). Because both *RTS1* overexpression and deletion of *HOG1* act as suppressors of *gcn5Δ* nocodazole sensitivity, we hypothesized that these opposing phenotypes may indicate that loss of Hog1 could rescue the *rts1Δ gcn5Δ* synthetic lethality. The triple mutant *hog1Δ rts1Δ gcn5Δ*, bearing a WT *URA3-GCN5* plasmid was constructed genetically. Triple mutants recovered from the cross were plated onto 5-FOA medium to determine if the WT *URA3-GCN5* plasmid could be lost. We found that the *hog1Δ rts1Δ gcn5Δ* was viable (Fig. 2b), indicating that concurrent deletion of *HOG1*, indeed, rescues the *rts1Δ gcn5Δ* synthetic lethality.

We next tested if loss of Hog1 kinase activity provided the specific mechanism for rescue of *rts1Δ gcn5Δ* synthetic lethality. In this case, the *CEN-HIS3-hog1-T174A* plasmid was transformed into the *CEN-URA3-GCN5-*bearing *hog1Δ rts1Δ gcn5Δ* strain. On 5-FOA medium, the *hog1Δ rts1Δ gcn5Δ* strain showed growth comparable to WT on 5-FOA medium, indicating that the catalytic inactivation of *HOG1* is what drives suppression of the *rts1Δ gcn5Δ* synthetic lethality (Fig. 2c). Further, the *hog1Δ rts1Δ gcn5Δ* mutant bearing the *CEN-HIS3-HOG1* plasmid showed compromised growth upon loss of the *CEN-URA3-GCN5* plasmid (Fig. 2d). Together, these results support the idea that loss of Hog1's catalytic activity, and not another property of the enzyme, is what rescues *rts1Δ gcn5Δ* synthetic lethality, pointing to a key enzymatic role in the Rts1–Gcn5 interaction.

### H2B threonine 91 is required for *hog1Δ* rescue of *gcn5Δ* nocodazole sensitivity

**Fig. 3.**
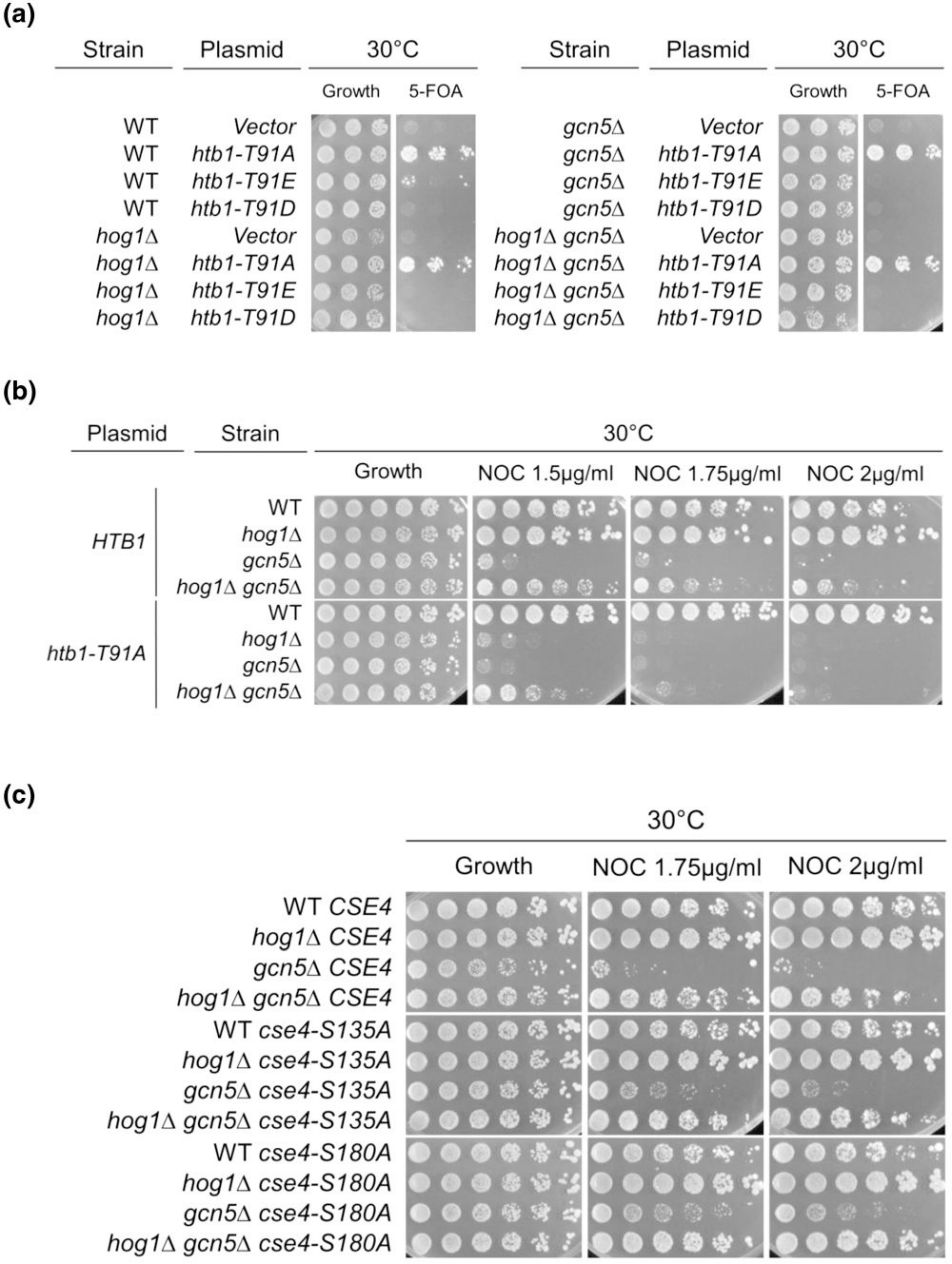
The histone H2B-T91A mutant abolishes *hog1Δ* rescue of *gcn5Δ* nocodazole sensitivity. a) Phosphomimetic H2B-T91E and H2B-T91D mutations cause lethality in all backgrounds. WT (LPY 14462), *hog1Δ* (LPY 23129), *gcn5Δ* (LPY 16434), and *hog1Δ gcn5Δ* (LPY 23128) strains were constructed, bearing a *URA3-HTB1* plasmid and each of three *HIS3- htb1* plasmids *(htb1-T94A, htb1-T91E,* or *htb1-T91D).* Strains were plated onto HIS-URA- (growth) and HIS- and incubated at 30°C for three days. The H2B-T91A mutation was found to be viable in all strains. b) *htb1-T91A* partially interferes with *hog1Δ* suppression of *gcn5Δ* nocodazole sensitivity. Strains from above with *HTB1* or *htb1-T91A* were plated onto HIS- (growth) and YPAD + NOC plates following overnight growth in HIS- media. The HIS- plate is used as a control to ensure retention of histone shuffle plasmid. Cells were incubated at 30°C for three days. In this case, *htb1-T91A* abolishes *hog1Δ* increased resistance to NOC in WT cells. c) Phosphodeficient *cse4-S180A* and *cse4-S135A* mutations have no negative effects on *hog1Δ gcn5Δ*. To test effects of the centromeric specific histone mutants, *cse4-S135A* and *cse4-S180A* WT strains were crossed with *hog1Δ gcn5Δ* (LPY 21366) to produce the *hog1Δ cse4* double mutant and the *hog1Δ gcn5Δ cse4* triple mutant. Strains were plated onto YPAD (Growth) and YPAD + NOC medium at 1.5 µg/ml, 1.75 µg/ml, 2 µg/ml, and 2.25 µg/ml concentrations. Cells were incubated at 30°C for three days. Here, *cse4-S180A* itself increases *gcn5Δ* nocodazole sensitivity, confirming previous results (Petty *et al.* 2018).

Our previous studies established that H2B-T91 plays an important role in the Gcn5–Rts1 interaction. This conserved residue is phosphorylated in vertebrates (phosphosite.com), and in yeast, the phosphodeficient H2B-T91A mutant hinders *RTS1* dosage-dependent rescue of *gcn5Δ* phenotypes (Petty *et al.* 2016). We tested if the same histone residue is involved in the Gcn5–Hog1 interaction. Confirming previous observations, we found that H2B-T91D/E phosphomimetic mutations cause lethality in all backgrounds (Fig. 3a). The *htb1-T91A* allele was found to hinder the growth of *hog1Δ gcn5Δ* growth on nocodazole-containing media, indicating that the phosphodeficient histone mutation partially abolishes the *hog1Δ* rescue of *gcn5Δ* sensitivity to nocodazole (Fig. 3b). Moderate rescue of *gcn5Δ* sensitivity to nocodazole remains in the *hog1Δ gcn5Δ htb1-T91A* strain at lower nocodazole concentrations (Fig. 3b). Together, these results confirmed that mutation of histone H2B-T91 exerts an effect on the Hog1–Gcn5 interaction, indicating that the phosphorylatable H2B-T91 residue plays an important role in the Hog1–Gcn5 interaction. Both Gcn5–Rts1 (Petty *et al.* 2016) and Gcn5–Hog1 (Fig. 3b) functional interactions rely on H2B-T91, further supporting the hypothesis that Hog1 and PP2A^Rts1^ together mediate dynamic histone modifications crucial for *gcn5Δ* mutants.

Beyond the canonical core histones, residues have been identified in the centromere-specific histone Cse4 that hinder *RTS1* dosage-dependent suppression of *gcn5Δ* DNA damage sensitivity but improve *gcn5Δ* resistance to nocodazole (Petty *et al.* 2018). Therefore, the effects of these, *cse4-S180A* and *cse4-S135A*, were tested in *hog1Δ gcn5Δ* mutants. Strains of *hog1Δ gcn5Δ* containing the integrated *cse4-S180A and cse4-S135A* alleles or WT *CSE4* were constructed and plated onto nocodazole-containing media. No difference was observed between the growth of the *hog1Δ gcn5Δ cse4-S135A*, *hog1Δ gcn5Δ cse4-S180A,* and *hog1Δ gcn5Δ CSE4* strains (Fig. 3c). Thus, we concluded that phosphodeficient *cse4* alleles do not negatively affect the Hog1–Gcn5 interaction.

### *hog1Δ* specifically restores *gcn5Δ* viability in response to nocodazole

**Fig. 4.**
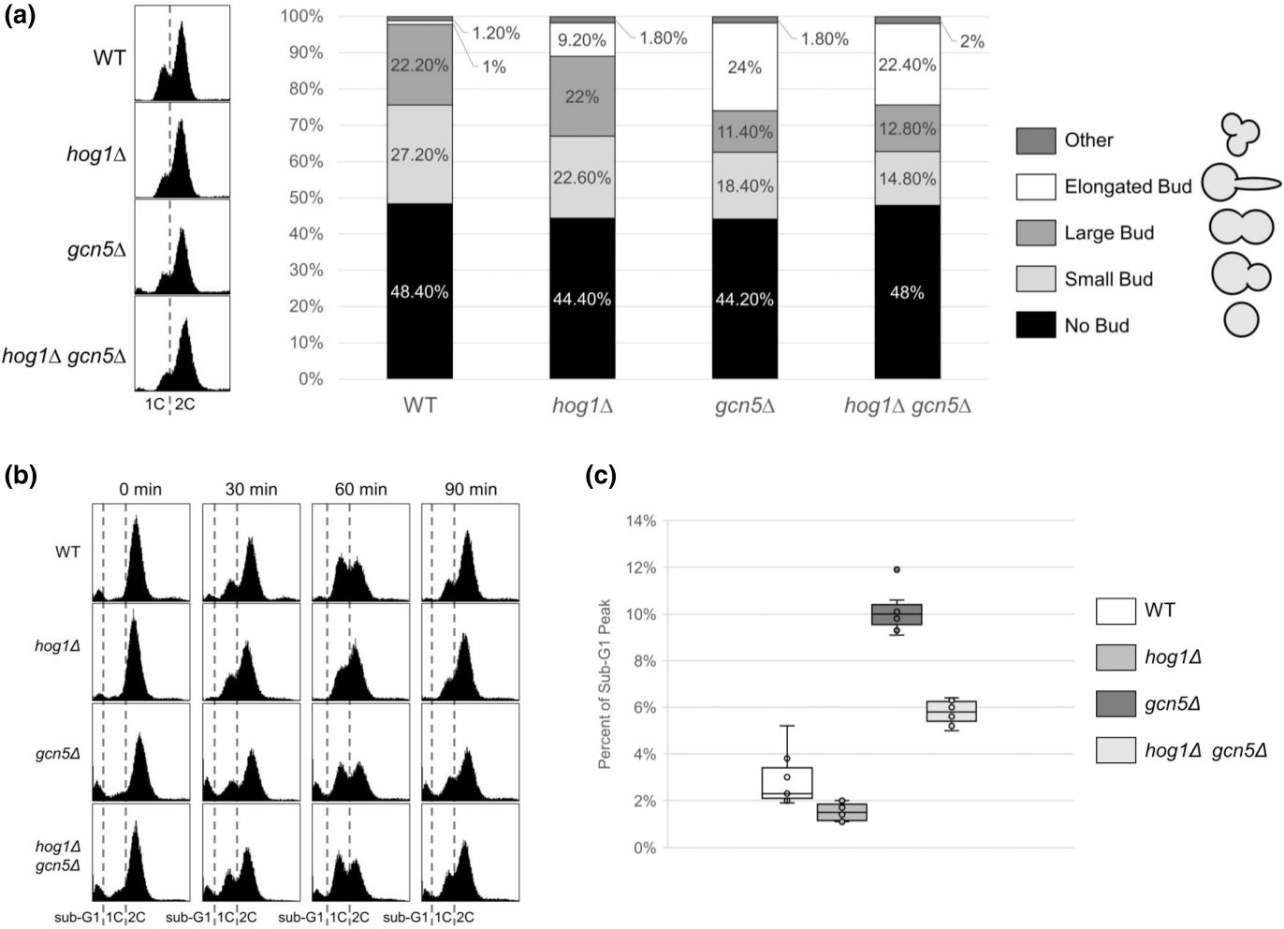
The *hog1Δ gcn5Δ* strain exhibits similar cell cycle progression and budding defects as *gcn5Δ* cells in asynchronous cell culture. a) Asynchronous cultures of WT (LPY 5), *hog1Δ* (LPY 21375), *gcn5Δ* (LPY 13319), *hog1Δ gcn5Δ* (LPY 21368) were prepared for flow cytometry (left). A total of 30,000 events for each were collected. 1C indicates 1 copy of the genetic material (G1 phase) with 2C indicating duplicated genomes (G2/M phase). *hog1Δ* cultures/populations have fewer cells in the G1 phase and an increased number of cells in the S phase. *gcn5Δ* and *hog1Δ gcn5Δ* showed similar slow G2/M progression. *The gcn5Δ* and *hog1Δ gcn5Δ* strains also showed an increased number of dead cells compared to WT and *hog1Δ*, indicated by the cell population located on the left corner of the display. Budding indices were also determined for 500 cells of each strain (right). The *hog1Δ* mutation alone caused a slight increase in the percentages of elongated buds, with *gcn5Δ* having an even larger increase. No significant differences of cells with different bud shapes were observed between *gcn5Δ*, and *hog1Δ gcn5Δ.* Data shown are representative of three independent experiments. b) Exponentially growing WT (LPY 5), *hog1Δ* (LPY 21375), *gcn5Δ* (LPY 13319), *hog1Δ gcn5Δ* (LPY 21368) cultures were treated with nocodazole (3.75 µg/ml for WT and *hog1Δ,* and 1.5 µg/ml for *gcn5Δ* and *hog1Δ gcn5Δ*. Note that strains deleted for *GCN5* are particularly sensitive, therefore must be treated with lower concentrations to avoid excessive cell death. These concentrations are not adequate to arrest WT cells). At *T* = 0 mins, cultures were released from the arrest, then samples taken every 30 mins and prepared for flow cytometry with a total of 30,000 events collected. Following nocodazole arrest and release, *hog1Δ gcn5Δ* shows a decreased sub-G1 population compared to *gcn5Δ.* Those cells with genetic content lower than 1C (fluorescence intensity < 800 AU) are denoted as sub-G1, likely corresponding to apoptotic or aneuploid cells. Whereas cell cycle progression between *hog1Δ gcn5Δ* and *gcn5Δ* is similar following nocodazole-induced mitotic arrest, a reduction in the sub-G1 population was observed in *hog1Δ gcn5Δ* when compared to *gcn5Δ*. c) Boxplots quantifying the percentages of the sub-G1 populations shown in (b). A lower percentage of sub-G1 cells was observed in *hog1Δ gcn5Δ* and *hog1Δ* strains when compared to *gcn5Δ* and WT. The statistical significance is *P*-value < 0.05 for all comparisons.

*RTS1* overexpression restores cell cycle progression in *gcn5Δ* mutants at both the G1/S transition and the transition between mitosis and G1 (Petty *et al.* 2016, 2018). Considering Hog1 as a new player in the Rts1–Gcn5 interaction, we hypothesized that deletion of *HOG1* rescues the *gcn5Δ* sensitivity to nocodazole by improving cell cycle progression. To test this possibility, asynchronous cell cultures of *gcn5Δ* and *hog1Δ gcn5Δ* were incubated overnight and diluted to 0.1 OD_600_ with fresh media and monitored after the resumption of exponential growth. The DNA content of these cell populations was evaluated via flow cytometry of propidium iodide-stained cells. Both *gcn5Δ* and *hog1Δ gcn5Δ* show an increased G2/M population compared to WT (Fig. 4a, left), indicating that under normal growth conditions, both *gcn5Δ* and *hog1Δ gcn5Δ* have defective cell cycle progression.

In *S. cerevisiae*, the budding cycle is directly related to the cell cycle (Zettel *et al.* 2003). Abnormal budding has been reported in *gcn5Δ* mutants in our earlier studies, along with its documentation in genome-wide studies and those directed at understanding *GCN5*'s role in centromere and kinetochore functions (Vernarecci *et al.* 2008; Watanabe *et al.* 2009; Petty *et al.* 2016). We evaluated budding phenotypes microscopically to determine the percentage of each cell type in an asynchronous cell culture. Unbudded cells, those with small buds, and those with large buds roughly correspond to populations undergoing G1, S, and G2/M phases of the cell cycle, respectively. Cells with elongated buds and other abnormal budding patterns likely result from disruption of the budding cycle or other mutant phenotypes. In WT cultures, unbudded cells and those with small buds and large buds are found at approximately 50, 25, and 25% of the total population, respectively (Fig. 4a, right). Only a small fraction of the WT population showed elongated (1%) or abnormal buds (1.2%). Compared to the WT*, gcn5Δ* and *hog1Δ gcn5Δ* cultures contained an increased proportion of elongated buds (24.0 and 22.4%) and decreased numbers of small and large buds (Fig. 4a, right), indicating that these two mutants exhibit similar budding defects in growing populations of cells.

We next tested if deletion of *HOG1* improves *gcn5Δ* progression through specific stages of the cell cycle. Mitotic arrest in growing cultures was induced by 1.5–3.75 µg/ml nocodazole. In this experiment, mitotic arrest in cells lacking *GCN5* was induced with a lower concentration of nocodazole to avoid excessive cell death due to the strain's sensitivity. After the cells were released from the arrest (0 mins), samples were collected and stained with propidium iodide for detection of DNA content. In *gcn5Δ* and *hog1Δ gcn5Δ* cultures, accumulation of cells in G1 at 60 mins and a decrease in the G2 population at 90 mins were observed (Fig. 4b). In both asynchronous and G2/M synchronized cell cultures, we have found no significant difference in the DNA content and budding index between *gcn5Δ* and *hog1Δ gcn5Δ* (Fig. 4a and b). These results indicate that under normal growth conditions in rich medium, *hog1Δ gcn5Δ* exhibit similar cell cycle defects as *gcn5Δ.* Thus, *hog1Δ* specifically rescues *gcn5Δ* sensitivity to end-point growth on nocodazole, without restoring normal cell cycle progression.

However, in the flow cytometry analysis, we noticed a significantly lower sub-G1 population in *hog1Δ gcn5Δ* compared to *gcn5Δ* following the nocodazole-induced mitotic arrest (Fig. 4b). When the percentage of the sub-G1 population is plotted at each time point, the median percentages of this population in *gcn5Δ* and *hog1Δ gcn5Δ* are approximately 10 and 6%, respectively (Fig. 4c). *hog1Δ* greatly decreases the percentage of sub-G1 cells following nocodazole-induced arrest in both WT and *gcn5Δ* backgrounds (Fig. 4c). These results suggest that although *hog1Δ* failed to improve *gcn5Δ* cell cycle progression in rich media, *hog1Δ* otherwise alleviates the negative effects caused by nocodazole arrest in the *gcn5Δ* mutant. Therefore, we suspected that *hog1Δ* might contribute to an increased resistance to nocodazole independently of a direct role in the regulation of cell cycle progression.

### *hog1Δ* plays a context-dependent role in *gcn5Δ* cell cycle progression

**Fig. 5.**
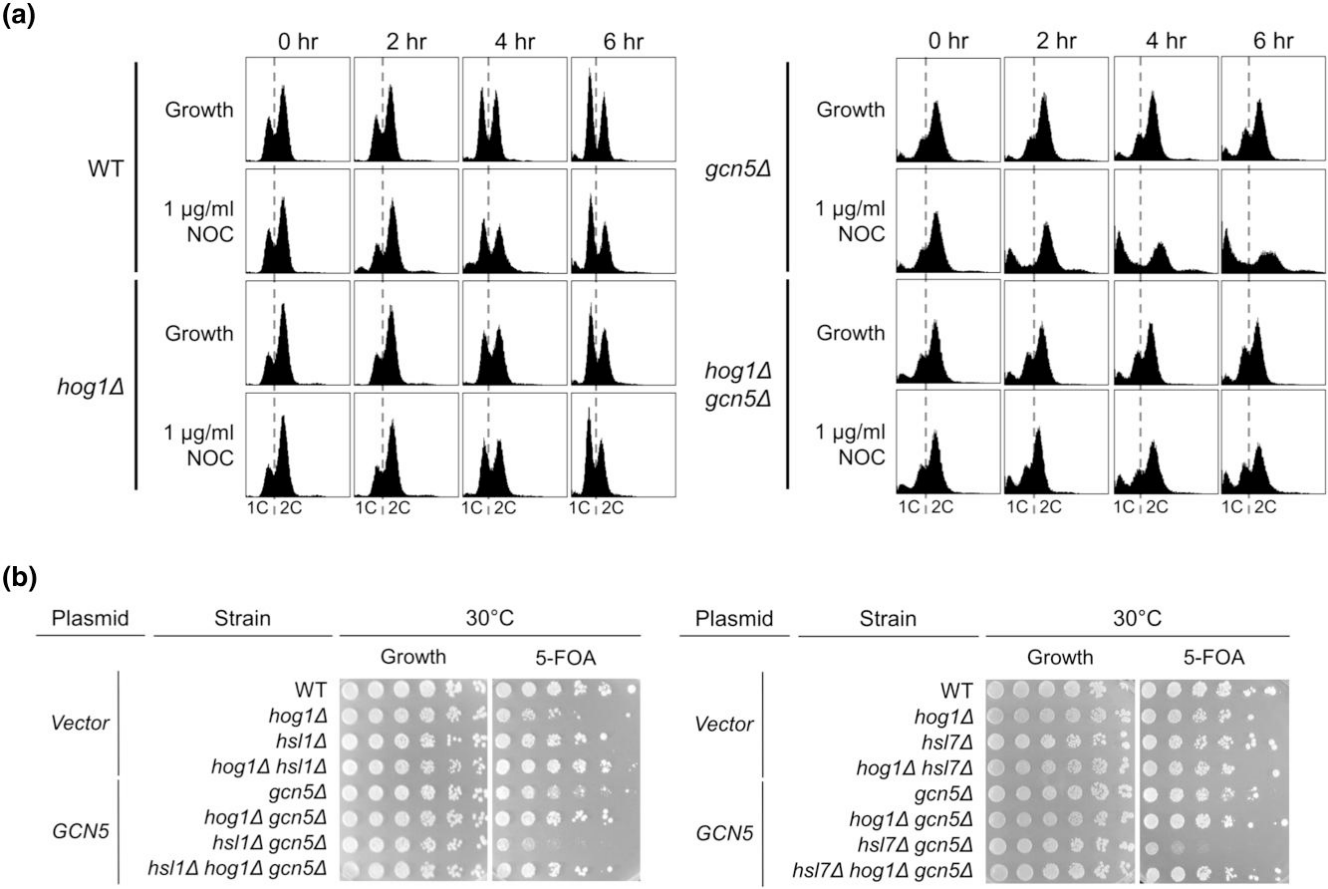
Diverse roles for *hog1Δ* in *gcn5Δ* in cell cycle regulation. a) Cultures of WT (LPY 5), *hog1Δ* (LPY 21375), *gcn5Δ* (LPY 13319), *hog1Δ gcn5Δ* (LPY 21368) cells were treated with nocodazole (1 µg/ml) or DMSO (solvent control) and incubated at 30˚C. *T* = 0 hour denotes the time at which nocodazole was added. Cultures and samples were collected and prepared for flow cytometry every hour. WT and *hog1Δ* show a similar pattern of cell cycle progression with marked improvement in cell cycle progression in *hog1Δ gcn5Δ* relative to *gcn5Δ* and a decrease in sub-1C and the >2C populations in *hog1Δ gcn5Δ.* b) Strains were constructed to probe for a role for *HOG1* in the morphogenesis checkpoint (Ruault and Pillus, 2006). WT (LPY 5)*, hog1Δ* (LPY 21375)*, hsl1Δ* (LPY 8937)*, hsl7Δ* (LPY 10892)*, hog1Δ hsl1Δ* (LPY 23191)*, hog1Δ hsl7Δ* (LPY 23194)*, gcn5Δ* (LPY 13319)*, hog1Δ gcn5Δ* (LPY 21367)*, hsl1Δ gcn5Δ* (LPY 9093)*, hsl1Δ hog1Δ gcn5Δ* (LPY 23192)*, hsl7Δ gcn5Δ* (LPY 8378)*, hsl7Δ hog1Δ gcn5Δ* (LPY 23193) were transformed with pLP1640 (*CEN*-*URA3*-*GCN5*) or pLP126 (*CEN-URA3-vector*) and plated on URA- (Growth) or 0.5 × 5-FOA. The *gcn5Δ hsl1Δ* and *gcn5Δ hsl7Δ* are synthetically lethal, whereas *hog1Δ hsl1Δ gcn5Δ* and *hog1Δ hsl7Δ gcn5Δ* triple mutants survive.

Nocodazole can be used in two different ways: to induce cell cycle arrest at the G2/M phase or to monitor the sensitivity of a strain to microtubule destabilization. Previous results showed that *hog1Δ gcn5Δ* cultures contained fewer sub-G1 cells in response to nocodazole-induced G2/M arrest. Based on these results, we hypothesized that deletion of *HOG1* specifically rescues growth of cells lacking *GCN5* in microtubule destabilizing conditions. Thus, using lower concentrations of nocodazole as a microtubule destabilizing agent, we tested if deletion of *HOG1* improves *gcn5Δ* cell cycle progression. Log-phase cultures growing in rich media were treated with nocodazole at 1 µg/ml, a concentration that does not induce cell cycle arrest, and were monitored for 6 hours following nocodazole addition. In asynchronous cultures, *gcn5Δ* cell cycle progression was disrupted, indicated by an increase in both the sub-G1 and greater than 2C populations (Fig. 5a). Deletion of *HOG1* in the *gcn5Δ* mutant resulted in a significant reduction in sub-G1 and greater 2C populations, consistent with a potential decrease in aneuploidy and apoptotic cells (Fig. 5a). Upon nocodazole treatment, *hog1Δ gcn5Δ* cultures exhibit an increased 2C population, indicating that cells are accumulating at G2/M (Fig. 5a). Similar increases in resistance to nocodazole and accumulation at G2/M were also observed in *hog1Δ* when compared to WT at 2 µg/ml nocodazole (not shown). These results indicate that loss of Hog1 improves cell cycle progression under microtubule destabilizing conditions.

Because deletion of *HOG1* improves *gcn5Δ* cell cycle progression under nocodazole conditions which hinder but do not halt mitotic progression, we tested if the Hog1–Gcn5 interaction might be further defined in the context of other cell cycle checkpoint functions.

In *S. cerevisiae*, the cell cycle and bud morphology are coupled by the morphogenesis checkpoint during the normal G2 phase. Hsl1, a key kinase regulator of the morphogenesis checkpoint, recruits Hsl7 and Swe1, a negative regulator of Cdc28, to the septin ring (Shulewitz *et al.* 1999). Hsl1-dependent Swe1 phosphorylation facilitates Swe1 degradation and cell cycle progression (McMillan *et al.* 1999). It has been observed that constitutive activation of the morphogenesis checkpoint resulting from deletion of either *HSL1* or *HSL7* is lethal in the *gcn5Δ* background (Ruault and Pillus, 2006). Deletion of the cyclin-dependent kinase inhibitor Swe1 alleviates the synthetic lethality in *gcn5Δ hsl1Δ* and *gcn5Δ hsl7Δ.* To test if loss of *HOG1* also rescues this lethality, the triple mutants *hog1Δ gcn5Δ hsl1Δ* and *hog1Δ gcn5Δ hsl7Δ* were constructed, initially supported with a *CEN-URA3-GCN5* plasmid. The mutants were then plated onto 5-FOA. Whereas *gcn5Δ hsl1Δ* and *gcn5Δ hsl7Δ* were inviable on 5-FOA, *hog1Δ gcn5Δ hsl1Δ* and *hog1Δ gcn5Δ hsl7Δ* showed growth comparable to WT strains (Fig. 5b). This suppression of *gcn5Δ*'s synthetic lethality with morphogenesis checkpoint mutants by *hog1Δ* suggests that the Hog1–Gcn5 interaction extends beyond the scope of mitosis and spindle assembly checkpoints.

Since both *hog1Δ* and *swe1Δ* rescue *gcn5Δ hsl1Δ* and *gcn5Δ hsl7Δ* synthetic lethality, these results indicate that in the context of the morphogenesis checkpoint, Hog1, like Swe1, may function in inhibiting cell cycle progression. Whereas in the context of the morphogenesis checkpoint, Hog1 plays a role in inhibiting the cell cycle via checkpoint activation, in the presence of nocodazole, deletion of *HOG1* seems to restore normal checkpoint function. Therefore, we conclude that *hog1Δ* plays a diverse and context-dependent role in *gcn5Δ* cell cycle progression.

## Discussion

Based on the previously established Gcn5–Rts1 functional interaction implicating H2B phosphorylation (Petty *et al.* 2016), we set out to determine if there were nuclear kinases which participated in the interaction, potentially through opposing the PP2A^Rts1^ contribution. Of the kinases screened, we found distinct patterns of suppression, and exacerbation of *gcn5Δ* phenotypes. Among the kinase mutants tested, *HOG1* was found specifically to suppress *gcn5Δ* sensitivity to the microtubule poison nocodazole as well as its synthetic lethality with *rts1Δ* (Fig. 2). Furthermore, we have identified distinct *HOG1*–*GCN5* interactions in the context of the spindle assembly checkpoint and the morphogenesis checkpoint. Like Gcn5 (Downey, 2021), Hog1 targets a large number of substrates active in a variety of cellular processes (Janschitz *et al.* 2019), thus it is likely that the context-dependency observed is caused by the collective effects exerted by multiple Hog1 substrates.

### A role for Hog1 in the Gcn5–PP2A Rts1 nexus

Hog1 plays a part in the Gcn5–Rts1 interaction in microtubule destabilizing conditions. To determine if phosphorylatable histone residues might be required in the Hog1–Gcn5 interaction, we focused on H2B-T91, previously found to be involved in the Rts1–Gcn5 interaction. In the *gcn5Δ hog1Δ* mutant, we discovered that the phosphodeficient *htb-T91A* mutation dampens the suppression of *gcn5Δ* sensitivity to nocodazole by *hog1Δ* (Fig. 3b). These results are consistent with potential joint roles for Hog1 and PP2A^Rts1^ in mediating dynamic phosphorylation on H2B-T91, which is crucial for the survival of *gcn5Δ* mutants.

We note that the sequence surrounding H2B-T91 does not meet the established Hog1 phosphorylation consensus, which ordinarily consists of a serine or a threonine preceding a proline residue (Mok *et al.* 2010). Thus, Hog1 may well activate another nuclear kinase, which in turn participates in the dynamic phosphorylation of H2B-T91. Indeed, H2B-T91 in yeast has been predicted as a target of Ste20, a member of the p21-activated kinase family (Petty *et al.* 2016, Wang *et al.* 2020). Additionally, Ste20 was found to mediate histone phosphorylation involved in a number of processes. Ste20-mediated H2B-S10 phosphorylation, which plays a role in apoptosis, was found to depend on the deacetylation of H2B-K11 by Hos3 (Ahn *et al.* 2005). Therefore, there is precedent for Ste20 histone phosphorylation function interacting with enzymes regulating histone acetylation. Furthermore, Ste20-mediated phosphorylation of H4-S47 plays a role in the modulation of osmotic stress-responsive genes (Viéitez *et al.* 2020). Previous studies have shown that Ste20 interacts with Hog1 by regulating the activation of Hog1 in response to osmotic stress (Raitt *et al.* 2000; O’Rourke and Herskowitz, 2002). One hypothesis is that deletion of Hog1 might lead to loss of Ste20-mediated histone phosphorylation, restoring the phosphorylation balance vital for the *gcn5Δ* mutant. However, loss of *STE20* instead was found to exacerbate *gcn5Δ* sensitivity to nocodazole (Fig. 1c), indicating that such a simple hypothesis does not capture the complete functional relationship between Hog1, Gcn5 and Ste20.

### Hog1 functionally opposes Gcn5 and Rts1 centromeric functions during the spindle assembly checkpoint

During investigation of the mechanism underlying the Hog1–Gcn5 interaction, we found that *hog1Δ* suppresses potential chromosome segregation defects as evidenced by fewer cells with abnormal DNA content in both WT and *gcn5Δ* backgrounds in microtubule destabilizing conditions (Figs. 4d and 5a). Furthermore, *hog1Δ gcn5Δ* cells contain increased G2/M populations under microtubule destabilizing conditions when compared to WT strains (Fig. 5a).

During the transition from metaphase to anaphase, proper attachment of spindle fibers to the kinetochore ensures faithful segregation of sister chromatids and prevents aneuploidy and cell death (Li and Murray, 1991). Nocodazole disrupts dynamic microtubule structures leading to activation of the spindle assembly checkpoint during mitosis (Vasquez *et al.* 1997; Blajeski *et al.* 2002). Low nocodazole concentrations mainly affect the spindle fiber interaction with the kinetochore without major structural changes to the spindle fibers (Wang and Burke, 1995). Therefore, we propose that under microtubule destabilizing conditions induced by the low dose 1 µg/ml nocodazole treatment, deletion of *HOG1* slows the kinetics of cell cycle progression, allowing for proper kinetochore-microtubule attachments to stabilize under otherwise less-than-ideal conditions. Accordingly, lower numbers of apoptotic and aneuploid cells were observed. The increased G2/M population in *hog1Δ gcn5Δ* further supports this interpretation.

In *S. cerevisiae*, anaphase onset and mitotic exit follow the faithful segregation of sister chromatids. The mitotic exit network (MEN) controls the completion of mitosis and cytokinesis by coordinating molecular events resulting in the inactivation of cyclin-dependent kinase (CDK), reversal of CDK-mediated phosphorylation, accumulation of CDK inhibitors, and promotion of cyclin destruction (Visintin *et al.* 1998; Jin *et al.* 2008; Campbell *et al.* 2019). Under conditions where the kinetochore-microtubule attachment is destabilized, proper MEN inhibition is required for appropriate spindle assembly checkpoint function to ensure correct orientation and segregation of sister chromatids (Caydasi *et al.* 2010). Reiser and colleagues (2006) reported that sustained activation of the HOG pathway rescues the mitotic exit defects in MEN mutants. The results reported here align with these observations in that no distinct improvement was observed in *hog1Δ gcn5Δ* cell cycle progression when compared to *gcn5Δ* under normal growth conditions. However, improvement in cell cycle progression was observed when nocodazole was introduced to growth media, akin to inhibiting mitotic exit. Furthermore, *hog1Δ* mutants showed a significant delay in the spindle disassembly checkpoint under normal growth condition (Pigula *et al.* 2014). Together, these results imply that in the context of mitosis, Hog1 may have a role in promoting mitotic progression and exit, acting in opposition to checkpoints that halt cell cycle progression. Based on our results and previous studies, we suspected that this specific function of Hog1 might be independent of the cell cycle modulation mediated by Hog1 upon osmotic shock. Indeed, Hog1 was found to delay mitotic exit by inhibiting anaphase onset during osmotic stress (Tognetti *et al.* 2020).

Previous studies have shown that Gcn5 plays an important role in the centromeric localization of Rts1, which in turn promotes the tension sensing function of the spindle assembly checkpoint (Petty *et al.* 2018). Since the spindle assembly checkpoint plays a crucial role in the survival of cells in microtubule destabilizing conditions, in preliminary studies we examined if *GCN5* had genetic relationships with the spindle assembly checkpoint mutants *bub1Δ* (Farr and Hoyt 1998; Musacchio 2015) and *mad2Δ* (Hardwick *et al.* 1999; Musacchio 2015) by testing the viability of *bub1Δ gcn5Δ* and *mad2Δ gcn5Δ* double mutants. No synthetically lethal interaction between the deletion of *GCN5* and the loss of Mad2 or Bub1 was observed. This result suggests that the Gcn5–Rts1 interaction and the spindle assembly checkpoint components Bub1 and Mad2 might function in distinct aspects of the spindle assembly checkpoint. Further investigation of the spindle assembly checkpoint is in order, including analysis of genetic interactions with other players (Musacchio 2015) or a future focus on their localization or phosphorylation in its regulation in *hog1Δ* and *gcn5Δ* mutants.

**Fig. 6.**
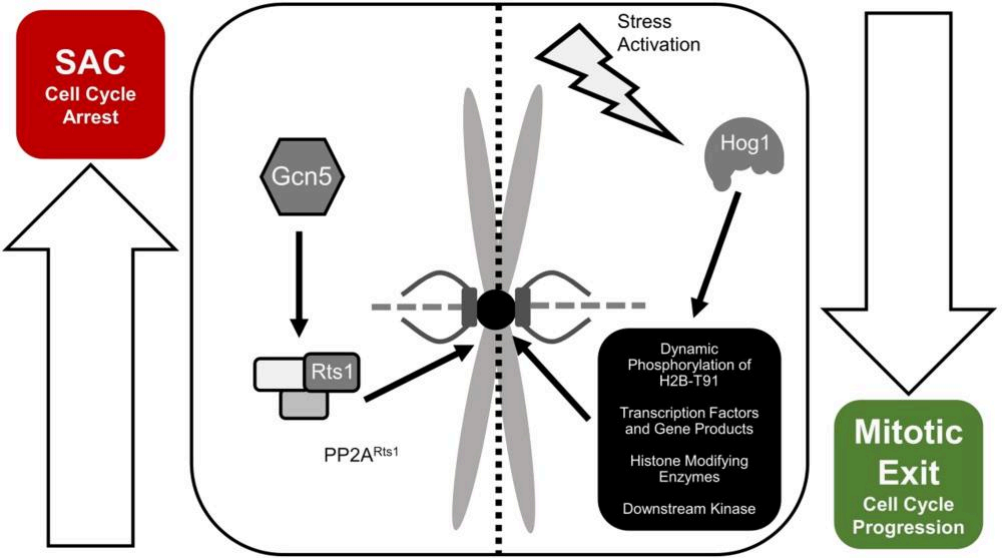
A functional tug of war between Hog1, Gcn5, and Rts1 in mitotic progression. Gcn5 promotes Rts1 localization to centromeres, which is crucial for the activation of the spindle assembly checkpoint (SAC). We propose that under microtubule destabilizing conditions, Gcn5 acts to promote Rts1 localization to the centromere, which results in spindle assembly checkpoint (SAC) activation and cell cycle arrest (Petty et al 2018). At the same time, activated Hog1 promotes mitotic exit via either direct interaction with the centromere or indirect mechanisms involving other interacting partners. We hypothesize that under such conditions, Hog1 functionally opposes Rts1 and Gcn5. A balance between Gcn5/Rts1-mediated SAC and Hog1-promoted mitotic exit ensures faithful segregation of chromosomes and undisturbed progression of the cell cycle. Such a balance between spindle assembly checkpoint activation and mitotic exit is crucial under microtubule destabilizing conditions. The balance between forces driving the SAC and mitotic exit result in *gcn5Δ* sensitivity to nocodazole. In *hog1Δ gcn5Δ,* deletion of *HOG1* causes a lack of driving force for mitotic exit during microtubule destabilizing conditions, restoring the balance between SAC activation and mitotic exit.

We hypothesize that Hog1 functionally opposes the Gcn5 and Rts1-mediated checkpoint. Upon microtubule destabilization, Hog1 may be activated to promote mitotic exit and oppose the cell cycle arrest induced by the spindle assembly checkpoint mediated by Gcn5 and PP2A^Rts1^ (Fig. 6). In WT cells, a balance between the effects imposed on the cell cycle by Gcn5/Rts1 and Hog1 ensures the cells undergo faithful chromosome segregation without prolonged mitotic stalling or arrest. However, in *gcn5Δ* mutants where the checkpoint is defective, Hog1-related induction of mitotic exit could exacerbate the defective checkpoint (Fig. 6). Thus, deletion of *HOG1* alleviates the chromosome segregation defect in *gcn5Δ* cells and increases the overall G2/M population when treated with nocodazole.

Previous studies demonstrated that a balance of dynamic phosphorylation at the centromere has a profound consequence on the interaction between the kinetochore and spindle fibers (Liu *et al.* 2010, Sherwin and Wang, 2019). One possibility is that Hog1 directly opposes the spindle assembly checkpoint and promotes mitotic exit by phosphorylating Cse4. A single threonine residue, Cse4-T133, aligns with the phosphorylation consensus site of Hog1. Hog1 and PP2A^Rts1^ thus potentially mediates a balance of phosphorylation at the centromere, modulating the spindle assembly checkpoint. Additionally, Hog1 might indirectly promote mitotic exit via interaction with cell cycle checkpoint components since Hog1 has been shown to mediate cell cycle arrest via modulation of cyclin expression and CDK inhibition under osmotic stress (Escoté *et al.* 2004; Clotet *et al.* 2006; Yaakov *et al.* 2009; Tognetti *et al.* 2020). Other studies have also shown that Hog1 recruits the histone deacetylase Rpd3 to osmotic stress gene promoters to modulate transcription of stress-responsive genes (De Nadal *et al.* 2004). Therefore, loss of Hog1 in *gcn5Δ* cells might restore the acetylation balance on key histone residues, thus improving *gcn5Δ* cell cycle progression.

### The many faces of *hog1Δ* in cell cycle progression in the absence of Gcn5

Beyond defective mitotic progression in *gcn5Δ* mutants, as evidenced by *gcn5Δ* sensitivity to the overexpression of Clb2 (Krebs *et al.* 2000), *gcn5Δ* disturbs other stages of cell cycle progression, including a significant reduction in the centromeric localization of Rts1 during the spindle assembly checkpoint, and slower progression of G1/S and G2/M (Petty *et al.* 2016, 2018). Based on the results from the present study, we propose that Hog1 plays different roles in *gcn5Δ* cell cycle progression based on its specific interaction partners and the context of the interaction. We hypothesize that Hog1 plays a role in promoting mitotic exit in its interaction with Gcn5 and Rts1 in the context of spindle assembly checkpoint. However, *hog1Δ* suppression of *gcn5Δ* synthetic lethality with morphogenesis checkpoint mutants suggests that in the context of that checkpoint, Hog1 plays a role in promoting cell cycle arrest. During the G2/M transition, timely Swe1 degradation depends on Hsl1 and Hsl7 and promotes the progression of the cell cycle. Loss of either Hsl1 or Hsl7 result in a constitutively activated morphogenesis checkpoint (McMillan *et al.* 1999). Previous studies identified deletion of *SWE1,* a gene encoding a CDK inhibitor that regulates G2/M progression, as a suppressor of *gcn5Δ* lethality under a constitutively activated morphogenesis checkpoint (Ruault and Pillus, 2006). Deletion of either *HOG1* or *SWE1* rescues *gcn5Δ hsl1Δ* and *gcn5Δ hsl7Δ* synthetic lethality, indicating that in the context of the morphogenesis checkpoint in *gcn5Δ* mutants, Hog1 and Swe1 play similar roles in the negative regulation of cell cycle progression. Indeed, upon sensing osmotic stress, Hog1 phosphorylates Hsl1, promoting Swe1 stabilization and G2/M arrest (Clotet *et al.* 2006). Together, these results suggest that there are multiple aspects of the Hog1–Gcn5 functional interaction contributing to cell cycle progression. The stage of the cell cycle, as well as the interacting partners, determines the role played by Hog1 and Gcn5, respectively, potentially as a function of their distinct substrates throughout the cell cycle.

In conclusion, through genetic analysis, we identified a previously unknown Hog1–Rts1–Gcn5 interaction in the context of histone modification and cell cycle progression. Our data support a hypothesis in which Hog1 and Rts1/Gcn5 functionally oppose each other at the spindle assembly checkpoint, pointing to a new role of the HOG pathway in the context of cell cycle checkpoint functions beyond classically defined responses to hyperosmolarity stress.

## Supplementary Material

jkad021_Supplementary_Data

## Data Availability

Yeast strains and plasmids will be provided upon request. We affirm that all data necessary for confirming the conclusions of the article are present within the article, figures, and tables. Supplemental tables detailing strains, plasmids, and oligonucleotides are available at *G3* online. Supplemental material available at G3 online.
