# Supplementary material for: Functional tug of war between kinases, phosphatases, and the Gcn5 acetyltransferase in chromatin and cell cycle checkpoint controls
Source: G3 (Bethesda). 2023 Feb 6;13(4):jkad021. doi: 10.1093/g3journal/jkad021 (PMC10085806; doi:10.1093/g3journal/jkad021)
Supplement: jkad021_Supplementary_Data [file jkad021_supplementary_data.zip › Table_S1_G3-2022-404014.docx]

Table S1 Strains used in this study.* All strains were constructed in this study or obtained from the lab collection.

| Strain | Genotype |
| --- | --- |
| LPY 5 | *MAT*a *ade2-1 can1-100 his3-11 leu2-3,112 trp1-1 ura3-1 GAL* |
| LPY 1873** | *MAT*a *ade2-1 can1-100 his3-11 leu2-3,112 trp1-1 ura3-1 GAL bar1 kss1∆::kanMX* |
| LPY 8156** | *MAT*a *ade2-1 can1-100 his3-11 leu2-3,112 trp1-1 ura3-1 GAL snf1∆::kanMX* |
| LPY 8378 | *MAT*a *ade2-1 can1-100 his3-11 leu2-3,112 trp1-1 ura3-1 GAL hsl7Δ::kanMX gcn5Δ::HIS3* |
| LPY 8653** | *MAT*a *ade2-1 can1-100 his3-11 leu2-3,112 trp1-1 ura3-1 GAL swe1∆::LEU2 gcn5∆:HIS3* |
| LPY 8937 | *MAT*a *ade2-1 can1-100 his3-11 leu2-3,112 trp1-1 ura3-1 GAL hsl1Δ::kanMX* |
| LPY 9093 | *MAT*a *ade2-1 can1-100 his3-11 leu2-3,112 trp1-1 ura3-1 GAL hsl1Δ::kanMX gcn5Δ::HIS3* |
| LPY 9400** | *MAT*a *ade2-1 can1-100 his3-11 leu2-3,112 trp1-1 ura3-1 GAL swe1∆::LEU2* |
| LPY 10796** | *MAT*a *ade2-1 can1-100 his3-11 leu2-3,112 trp1-1 ura3-1 GAL ste20Δ::kanMX* |
| LPY 10802** | *MAT*a *ade2-1 can1-100 his3-11 leu2-3,112 trp1-1 ura3-1 GAL ste20Δ::kanMX gcn5Δ::HIS3* |
| LPY 10892 | *MAT*a *ade2-1 can1-100 his3-11 leu2-3,112 trp1-1 ura3-1 GAL hsl7Δ::HIS3* |
| LPY 13319 | *MAT*a *ade2-1 can1-100 his3-11 leu2-3,112 trp1-1 ura3-1 GAL gcn5∆::natMX* |
| LPY 13440** | *MAT*a *ade2-1 can1-100 his3-11 leu2-3,112 trp1-1 ura3-1 GAL snf1∆::TRP1 gcn5∆::natMX* |
| LPY 14461 | *MAT*α *ade2-1 can1-100 his3-11 leu2-3,112 trp1-1 ura3-1 GAL hht1-hhf1Δ::kanMX hta1-htb1Δ::natMX hta2-htb2Δ::HPH* |
| LPY 14462 | *MAT*a *ade2-1 can1-100 his3-11 leu2-3,112 trp1-1 ura3-1 GAL hht1-hhf1Δ::kanMX hta1-htb1Δ::natMX hta2-htb2Δ::HPH* |
| LPY 15178 | *MAT*a *ade2-1 can1-100 his3-11 leu2-3,112 trp1-1 ura3-1 GAL rts1Δ::kanMX gcn5Δ:natMX* |
| LPY 16356** | *MAT*a *ade2-1 can1-100 his3-11 leu2-3,112 trp1-1 ura3-1 GAL sch9∆::kanMX* |
| LPY 16434 | *MAT*a *ade2-1 can1-100 his3-11 leu2-3,112 trp1-1 ura3-1 GAL hht1-hhf1Δ::kanMX hta1-htb1Δ::natMX hta2-htb2Δ::HPH gcn5Δ::natMX* |
| LPY 17138** | *MAT*a *ade2-1 can1-100 his3-11 leu2-3,112 trp1-1 ura3-1 GAL sch9∆::kanMX gcn5∆::natMX* |
| LPY 18171** | *MAT*a *ade2-1 can1-100 his3-11 leu2-3,112 trp1-1 ura3-1 GAL kss1∆::HIS3 gcn5∆::natMX* |
| LPY 18427** | *MAT*a *ade2-1 can1-100 his3-11 leu2-3,112 trp1-1 ura3-1 GAL pho85∆::kanMX* |
| LPY 18491** | *MAT*a *ade2-1 can1-100 his3-11 leu2-3,112 trp1-1 ura3-1 GAL pho85∆::kanMX gcn5∆::natMX* |
| LPY 19785** | *MAT*a *ade2-1 can1-100 his3-11 leu2-3,112 trp1-1 ura3-1 GAL tel1∆::kanMX* |
| LPY 20086** | *MAT*a *ade2-1 can1-100 his3-11 leu2-3,112 trp1-1 ura3-1 GAL chk1∆::kanMX* |
| LPY 20132** | *MAT*a *ade2-1 can1-100 his3-11 leu2-3,112 trp1-1 ura3-1 GAL tel1∆::kanMX gcn5∆::natMX* |
| LPY 20368** | *MAT*a *ade2-1 can1-100 his3-11 leu2-3,112 trp1-1 ura3-1 GAL ctk1∆::kanMX* |
| LPY 20227** | *MAT*a *ade2-1 can1-100 his3-11 leu2-3,112 trp1-1 ura3-1 GAL dun1∆::kanMX* |
| LPY 20298** | *MAT*a *ade2-1 can1-100 his3-11 leu2-3,112 trp1-1 ura3-1 GAL chk1∆::kanMX gcn5∆::natMX* |
| LPY 20366** | *MAT*a *ade2-1 can1-100 his3-11 leu2-3,112 trp1-1 ura3-1 GAL ctk1∆::kanMX gcn5∆::natMX* |
| LPY 20370** | *MAT*a *ade2-1 can1-100 his3-11 leu2-3,112 trp1-1 ura3-1 GAL dun1∆::kanMX gcn5∆::natMX* |
| LPY 20612 | *MAT*a *ade2-1 can1-100 his3-11 leu2-3,112 trp1-1 ura3-1 GAL (HA)3-(HIS)6-cse4-S135A* |
| LPY 20780 | *MAT*a *ade2-1 can1-100 his3-11 leu2-3,112 trp1-1 ura3-1 GAL gcn5∆::natMX (HA)3-(HIS)6-cse4-S180A* |
| LPY 20786 | *MAT*a *ade2-1 can1-100 his3-11 leu2-3,112 trp1-1 ura3-1 GAL (HA)3-(HIS)6-CSE4* |
| LPY 20788 | *MAT*a *ade2-1 can1-100 his3-11 leu2-3,112 trp1-1 ura3-1 GAL gcn5∆::natMX (HA)3-(HIS)6-CSE4* |
| LPY 20801 | *MAT*a *ade2-1 can1-100 his3-11 leu2-3,112 trp1-1 ura3-1 GAL gcn5∆::natMX (HA)3-(HIS)6-cse4-S135A* |
| LPY 20827 | *MAT*a *ade2-1 can1-100 his3-11 leu2-3,112 trp1-1 ura3-1 GAL (HA)3-(HIS)6-cse4-S180A* |
| LPY 21366 | *MAT*α *ade2-1 can1-100 his3-11 leu2-3,112 trp1-1 ura3-1 GAL hog1∆::kanMX gcn5∆:: natMX* |
| LPY 21367 | *MAT*a *ade2-1 can1-100 his3-11 leu2-3,112 trp1-1 ura3-1 GAL hog1∆::kanMX gcn5∆:: natMX* |
| LPY 21368 | *MAT*a *ade2-1 can1-100 his3-11 leu2-3,112 trp1-1 ura3-1 GAL hog1∆::kanMX gcn5∆:: natMX* |
| LPY 21375 | *MAT*a *ade2-1 can1-100 his3-11 leu2-3,112 trp1-1 ura3-1 GAL hog1∆::kanMX* |
| LPY 21648** | *MAT*a *ade2-1 can1-100 his3-11 leu2-3,112 trp1-1 ura3-1 GAL cka1∆::kanMX* |
| LPY 21650** | *MAT*a *ade2-1 can1-100 his3-11 leu2-3,112 trp1-1 ura3-1 GAL cka2∆::kanMX* |
| LPY 21669** | *MAT*a *ade2-1 can1-100 his3-11 leu2-3,112 trp1-1 ura3-1 GAL cka1∆::kanMX gcn5∆::natMX* |
| LPY 21673** | *MAT*a *ade2-1 can1-100 his3-11 leu2-3,112 trp1-1 ura3-1 GAL cka2∆::kanMX gcn5∆::natMX* |
| LPY 23128 | *MAT*a *ade2-1 can1-100 his3-11 leu2-3,112 trp1-1 ura3-1 GAL hht1-hhf1Δ::kanMX hta1-htb1Δ::natMX hta2-htb2Δ::HPH hog1Δ::kanMX gcn5Δ::natMX* |
| LPY 23129 | *MAT*a *ade2-1 can1-100 his3-11 leu2-3,112 trp1-1 ura3-1 GAL hht1-hhf1Δ::kanMX hta1-htb1Δ::natMX hta2-htb2Δ::HPH hog1Δ::kanMX* |
| LPY 23135 | *MAT*a *ade2-1 can1-100 his3-11 leu2-3,112 trp1-1 ura3-1 GAL hog1∆::kanMX gcn5∆::natMX (HA)3-(HIS)6-cse4-S180A* |
| LPY 23136 | *MAT*a *ade2-1 can1-100 his3-11 leu2-3,112 trp1-1 ura3-1 GAL hog1∆::kanMX gcn5∆::natMX (HA)3-(HIS)6-CSE4* |
| LPY 23137 | *MAT*a *ade2-1 can1-100 his3-11 leu2-3,112 trp1-1 ura3-1 GAL hog1∆::kanMX gcn5∆::natMX (HA)3-(HIS)6-cse4-S135A* |
| LPY 23138 | *MAT*a *ade2-1 can1-100 his3-11 leu2-3,112 trp1-1 ura3-1 GAL hog1∆::kanMX (HA)3-(HIS)6-cse4-S180A* |
| LPY 23139 | *MAT*a *ade2-1 can1-100 his3-11 leu2-3,112 trp1-1 ura3-1 GAL hog1∆::kanMX (HA)3-(HIS)6-CSE4* |
| LPY 23140 | *MAT*a *ade2-1 can1-100 his3-11 leu2-3,112 trp1-1 ura3-1 GAL hog1∆::kanMX (HA)3-(HIS)6-cse4-S135A* |
| LPY 23147 | *MAT*a *ade2-1 can1-100 his3-11 leu2-3,112 trp1-1 ura3-1 GAL hog1-T174A gcn5∆::natMX* |
| LPY 23150 | *MAT*a *ade2-1 can1-100 his3-11 leu2-3,112 trp1-1 ura3-1 GAL hog1-T174A* |
| LPY 23189 | *MAT*a *ade2-1 can1-100 his3-11 leu2-3,112 trp1-1 ura3-1 GAL hog1∆::kanMX gcn5∆::natMX rts1∆::kanMX* |
| LPY 23191 | *MAT*a *ade2-1 can1-100 his3-11 leu2-3,112 trp1-1 ura3-1 GAL hog1Δ::kanMX hsl1Δ::kanMX* |
| LPY 23192 | *MAT*a *ade2-1 can1-100 his3-11 leu2-3,112 trp1-1 ura3-1 GAL hsl1Δ::kanMX hog1Δ::kanMX gcn5Δ::natMX* |
| LPY 23193 | *MAT*a *ade2-1 can1-100 his3-11 leu2-3,112 trp1-1 ura3-1 GAL hsl7Δ::HIS3 hog1Δ::kanMX gcn5Δ::natMX* |
| LPY 23194 | *MAT*a *ade2-1 can1-100 his3-11 leu2-3,112 trp1-1 ura3-1 GAL hog1Δ::kanMX hsl7Δ::HIS3* |

*Note that this list includes strains noted specifically in the text and also those single and double mutants (**) that were used in experiments summarized in Figure 1(c), but which are not designated specifically in the text.
