# Supplementary material for: Functional tug of war between kinases, phosphatases, and the Gcn5 acetyltransferase in chromatin and cell cycle checkpoint controls
Source: G3 (Bethesda). 2023 Feb 6;13(4):jkad021. doi: 10.1093/g3journal/jkad021 (PMC10085806; doi:10.1093/g3journal/jkad021)
Supplement: jkad021_Supplementary_Data [file jkad021_supplementary_data.zip › Table_S2_G3-2022-404014.docx]

Table S2 Plasmids included in the study. Unless otherwise noted, plasmids were constructed in this study or obtained from the lab collection.

| Plasmid | Genotype | Source |
| --- | --- | --- |
| pLP 60 | *CEN-HIS3-Vector* |  |
| pLP 126 | *CEN-URA3-Vector* |  |
| pLP 1640 | *CEN-URA3-GCN5* |  |
| pLP 2212 | *CEN-URA3-HTA1-HTB1-HHT1-HHF1* | M.M. Smith |
| pLP 2482 | *CEN-HIS3-HTA1-htb1-T91A-FLAG* |  |
| pLP 2492 | *CEN-HIS3-HTA1-HTB1-FLAG* |  |
| pLP 2689 | *CEN-HIS3-HTA1-htb1-T91E-FLAG* |  |
| pLP 2770 | *CEN-HIS3-HTA1-htb1-T91D-FLAG* |  |
| pLP 3508 | *CEN-URA3-CAS9* | L. McDonnell |
| pLP 3512 | *CEN-HIS3-HOG1* |  |
| pLP 3513 | *CEN-HIS3-hog1-T174A* |  |
