# Supplementary material for: Functional tug of war between kinases, phosphatases, and the Gcn5 acetyltransferase in chromatin and cell cycle checkpoint controls
Source: G3 (Bethesda). 2023 Feb 6;13(4):jkad021. doi: 10.1093/g3journal/jkad021 (PMC10085806; doi:10.1093/g3journal/jkad021)
Supplement: jkad021_Supplementary_Data [file jkad021_supplementary_data.zip › Table_S3_G3-2022-404014.docx]

Table S3 Oligonucleotides included in the study.

| Oligo | Gene | Use | Sequence |
| --- | --- | --- | --- |
| oLP 2362 | *HOG1* | Cloning | GGTAGCCCTTCATTACGGCATAACG |
| oLP 2363 | *HOG1* | Cloning and Sequencing | CCTTTTTCTTCCAGTTTTACTAGTAAATCCAATGCGG |
| oLP 2491 | *HOG1* | Sequencing | GCTCAGCCACGGACACTTTG |
| oLP 2506 | *HOG1* | Mutagenesis | GAATTCAAGACCCTCAAATGGCAGGCTATGTTTCCAC |
| oLP 2507 | *HOG1* | Mutagenesis | GTGGAAACATAGCCTGCCATTTGAGGGTCTTGAATTC |
| oLP 2508 | *HOG1* | gRNA | /5Phos/GATCAGGTGCCCTGTAGTATCTAGGTTTTAGAGCTAG |
| oLP 2509 | *HOG1* | gRNA | /5Phos/CTAGCTCTAAAACCTAGATACTACAGGGCACCT |
| oLP 2510 | *HOG1* | HDR | CGGTCTAGCAAGAATTCAAGACCCTCAAATGGCAGGCTATGTTTCTACTAGATACTACAG |
| oLP 2511 | *HOG1* | HDR | CGTCATATTTTTGCCACGTTAGCATGATTTCAGGTGCCCTGTAGTATCTAGTAGAAAC |
